# Salivary microbiota of periodontitis aggravates bone loss in ovariectomized rats

**DOI:** 10.3389/fcimb.2022.983608

**Published:** 2022-08-12

**Authors:** Nannan Wang, Lichun Zheng, Jun Qian, Min Wang, Lili Li, Yuezhen Huang, Qian Zhang, Yanfen Li, Fuhua Yan

**Affiliations:** Department of General Dentistry, Nanjing Stomatological Hospital, Medical School of Nanjing University, Nanjing, China

**Keywords:** periodontitis, osteoporosis, salivary microbiota, gut microbiota, bone loss

## Abstract

The mechanisms underlying the crosstalk between periodontitis and osteoporosis remain unclear. Recently, the gut microbiota has been recognized as a pivotal regulator of bone metabolism, and oral and gut mucosae are microbiologically connected. In this study, we investigated the effects of periodontitis on osteoporosis through the oral-gut axis. The salivary microbiota of patients with periodontitis was collected and then pumped into the intestine of Sprague–Dawley rats *via* intragastric administration for 2 weeks. An osteoporosis model was established using ovariectomy. Changes in the maxillae and femora were evaluated using microcomputed tomography (micro CT) and HE staining. Intestinal barrier integrity and inflammatory factors were examined using real-time quantitative polymerase chain reaction and immunofluorescence. The gut microbiota was profiled by 16S rRNA gene sequencing. Metabolome profiling of serum was performed using liquid chromatography-mass spectrometry sequencing. Micro CT and HE staining revealed osteoporotic phenotypes in the maxillae and femora of ovariectomized (OVX) rats. Our results confirmed that the salivary microbiota of patients with periodontitis aggravated femoral bone resorption in OVX rats. In addition, intestinal inflammation was exacerbated after periodontitis salivary microbiota gavage in OVX rats. Correlation analysis of microbiota and metabolomics revealed that lipolysis and tryptophan metabolism may be related to the bone loss induced by the salivary microbiota of patients with periodontitis. In conclusion, periodontitis can aggravate long bone loss through the oral-gut axis in OVX rats.

## Introduction

Periodontitis is a chronic inflammatory disease caused by dysbiosis of the periodontal microbiota, resulting in resorption of alveolar bone and destruction of the periodontal ligament. The alveolar bone is responsive to the oral microenvironment and influenced by systemic metabolism (Hathaway-Schrader and Novince, 2021). Osteoporosis is characterized by low bone mass and deterioration of the trabecular microstructure, which often occurs in elderly individuals, especially postmenopausal women. Previous studies have revealed a positive association between periodontitis and osteoporosis (Luo et al., 2014; Xu et al., 2021); however, the mechanism by which periodontitis influences systemic skeletal metabolism remains unclear.

The gut microbiota has emerged as an important regulatory factor of bone metabolism (Chen et al., 2017; Li et al., 2019; Chevalier et al., 2020) and is necessary for the pathological process of bone loss in ovariectomized (OVX) animal models (Yuan et al., 2022). A large population-based study showed that the genera *Bacteroides*, *Blautia*, *Phascolarctobacterium*, *Oscillospira*, *Ruminococcaceae* and *Actinobacillus* were elevated in osteoporosis (Ling et al., 2021). Probiotics such as *Lactobacillus reuteri* and *Lactobacillus rhamnosus* significantly decrease the intestinal permeability and prevent bone loss (Britton et al., 2014; Li et al., 2016). Furthermore, metabolites produced by the gut microbiota—such as short-chain fatty acids, amino acids, and polyamines—are related to the change of systemic bone mass (Lucas et al., 2018; Chevalier et al., 2020; Ling et al., 2021).

The oral cavity, which is the entry site into the gastrointestinal tract, harbors diverse microbial communities (Peng et al., 2022), and previous reports support the hypothesis that oral and gastrointestinal mucosae are microbiologically connected (Read et al., 2021; Bao et al., 2022). In addition, the salivary microbiota of periodontitis, which is rich in oral pathobionts, can translocate from the oral cavity to the intestine and disrupt the colonization resistance of gut-resident microbiota (Kitamoto et al., 2020). Our previous study demonstrated that treatment with the periodontitis salivary microbiota can lead to gut dysbiosis and worsen colitis (Qian et al., 2022a). Therefore, we speculate that the oral-gut axis might play a pivotal role in periodontitis affecting the systemic diseases.

Currently, studies on the salivary microbiota in periodontitis have mainly adopted the method of single-strain or *in vitro* culture. To better simulate the complex oral microbiota in periodontitis, we collected salivary microbiota from patients with periodontitis. Subsequently, we explored the effect of the periodontitis salivary microbiota on bone mass in rats with OVX-induced osteoporosis, where the potential biological pathways—including the gut barrier, microbiota, and metabolic profiling—were also explored (**Supplementary Figure S1**).

## Materials and methods

### Patient saliva collection and preparation

Patients diagnosed with periodontitis were recruited from the Nanjing Stomatological Hospital, Medical School of Nanjing University (NJSH-2021NL-93). The inclusion criteria were as follows: (1) patients diagnosed with Stage III or IV periodontitis based on the new classification proposed in the 2017 AAP and EAP World Workshop, and (2) patients over the age of 18. The exclusion criteria were set as follows: (1) patients who had received periodontal therapy in the last 12 months; (2) patients who had received medication in the last 6 months; and (3) patients with systemic disease, psychiatric disorders, pregnancy or lactation, and other oral diseases that might affect the periodontal status. All the included patients were confirmed by an experienced periodontist. A total of 18 eligible patients were recruited for the present study.

Unstimulated salivary samples were collected and analyzed using a previously described method (Atarashi et al., 2017). Briefly, saliva samples were mixed with an equal volume (w/v) of phosphate-buffered saline (PBS) containing 20% glycerol/PBS, later snap-frozen in liquid nitrogen, and then stored at –80°C until use.

### Animals and experimental design

The experimental animal protocol was approved by the ethics authorities of Nanjing Agriculture University (PZW2021026). Thirty-two female Sprague-Dawley rats (9-week-old) were purchased and kept in a specific pathogen-free facility. After a 3-week adaptation period, the rats were anesthetized and either sham-operated (Sham) or bilaterally ovariectomized (OVX). At 10 weeks after the operation, the rats were gavaged with PBS or the salivary microbiota of patients with periodontitis. Before the intragastric administration, the frozen salivary samples were thawed, centrifuged at 3,300 × *g* for 10 min at 4°C, and suspended in an equal volume of PBS. The saliva samples from different patients were well mixed when used. Each rat was given 1 mL of liquid (PBS or saliva) every other day for 2 weeks. All rats were then sacrificed, and samples were collected for subsequent tests. The rats were randomly and equally allocated to the following groups:

1. ShamPBS group: sham-operated and PBS gavaged;
2. ShamSP group: sham-operated and gavaged with the salivary microbiota of patients with periodontitis;
3. OVXPBS group: ovariectomized and PBS gavaged;
4. OVXSP group: ovariectomized and gavaged with the salivary microbiota of patients with periodontitis.

### Microcomputed tomography analysis

Femora and maxillae dissected from rats were fixed in 4% paraformaldehyde for 72 h and scanned using micro-computed tomography (micro-CT) with a Skyscan 1176 scanner (Bruker, Karlsruhe, Germany). Data Viewer and CTAn software were used to analyze bone parameters. Bone mineral density (BMD) was obtained using two hydroxyapatite [Ca_10_(PO_4_)_6_(OH)_2_] phantoms with BMD values of 0.250 and 0.750 g/cm^3^ as a reference.

The region of interest for the femoral trabecular was set at 1.00 from the distal growth plate level, extending toward the proximal femora (112 slides). For maxillae, trabecular morphometry was measured in the furcation area of the left maxillary second molar root, as previously described, with minor modifications (Dai et al., 2014; Johnston and Ward, 2015). The trabecular parameters of bone volume versus total volume ratio (BV/TV), trabecular thickness (Tb.Th), trabecular spacing (Tb.Sp), and trabecular number (Tb.N) were measured and quantified.

### Histological analysis

Femora and maxillae were decalcified and embedded in paraffin blocks. Sections were prepared for hematoxylin and eosin (HE) staining. PANNORAMIC MIDI (3DHISTECH Ltd., Budapest, Hungary) and CaseViewer software were used to scan and evaluate the sections.

### Tartrate-resistant acid phosphatase staining

The number of osteoclasts was quantified using tartrate-resistant acid phosphatase (TRAP) staining of femurs. The sections were evaluated using the CaseViewer software. The osteoclast numbers were calculated by averaging each section’s four different visual regions in each section.

### Immunofluorescence analysis

Immunofluorescent staining was performed to determine the expression of zona occludens protein 1 (ZO-1) in the colon. Briefly, sections were incubated with rabbit anti-ZO-1 (Servicebio, Wuhan, China) overnight at 4°C and incubated with a cyanine 3-conjugated goat anti-rabbit IgG secondary antibody (Servicebio, Wuhan, China). Nuclei were then stained with 4′,6-diamidino-2-phenylindole for 10 min. A confocal laser microscope (NikonA1, Nikon Inc., Tokyo, Japan) was used to obtain the images.

### Serum analysis

The levels of high-density lipoprotein cholesterol (HDL-C) (Fosun Diagnostics, Shanghai, China) and low-density lipoprotein cholesterol (LDL-C) (Fosun Diagnostics, Shanghai, China) in serum were assayed using a Siemens ADVIA 1800 Automated Chemistry Analyzer. In addition, serum levels of CTX-I (Cusabio, Wuhan, China) were measured using enzyme-linked immunosorbent assay (ELISA) according to the manufacturer’s instructions.

### Colonic RNA preparation and quantitative polymerase chain reaction

RNA was extracted from colonic tissues using the RNAprep Pure Tissue Kit (Tiangen, Beijing, China) and then reverse-transcribed into cDNA using the HiScript III RT SuperMix (Vazyme, Nanjing, China). An ABI ViiA 7 detection system (Thermo Fisher Scientific, Waltham, USA) was used for quantitative polymerase chain reaction (qPCR) following the manufacturer’s instructions. All reactions were carried out in triplicate. Analysis was performed using the 2^−ΔΔCt^ method. The β-actin was set as the housekeeping gene. The primers used in this study are listed in **Supplementary Table S1**.

### Gut microbiota analysis

Cecal samples were collected after sacrificing the rats, then snap-frozen and stored at –80°C. 16S rRNA sequencing was conducted by OeBiotech (Shanghai, China). Briefly, microbial DNA was isolated from cecum samples using MagPure Soil DNA LQ Ki (Magen, Guangzhou, China). The V3-V4 region of the 16S rRNA gene was amplified. Sequencing was performed using an Illumina NovaSeq6000 system (Illumina Inc., CA, USA). The original two-terminal sequence was dehybridized using Trimmomatic software. Clean reads were subjected to primer sequence removal and clustering to generate operational taxonomic units (OTUs) using the VSEARCH software with a threshold of 97% similarity. All representative reads were assigned to a taxon using the Ribosomal Database Project classifier (confidence threshold of 70%) against the Silva database (version 132). The α/β indices were calculated using QIIME (Version 1.8.0).

### Metabolome profiling of serum

Metabolome profiling of serum was performed using chromatography-mass spectrometry (LC-MS) sequencing, as previously described (Zhang P. et al., 2021). Data were processed using Progenesis QI V2.3 (Nonlinear). Online databases, including the Human Metabolome Database, Metlin, Electron Microscopy Data Bank, Lipidmaps (V2.3), and Protein Model DataBase, were used to qualify the data. Orthogonal partial least squares discriminant analysis (OPLS-DA) was used to identify the metabolites of different groups and obtain variable importance of projection (VIP) values. The pathways involved in the differential metabolites were annotated using the metabolic pathways in the Kyoto Encyclopedia of Genes and Genomes (KEGG) database (https://www.kegg.jp).

### Statistics

The statistical analyses were carried out by GraphPad Prism 9 unless otherwise specified. Differences between two groups were evaluated using a two-tailed Student’s *t*-test (parametric) or the Mann-Whitney U test (nonparametric). For more than two groups, one-way analysis of variance (ANOVA; parametric) or the Kruskal–Wallis test (nonparametric) was performed, followed by Bonferroni’s multiple comparisons test. A value of *p* < 0.05 was considered significantly significant.

## Results

### OVX reduces bone density of alveolar bone

**Figure 1:**
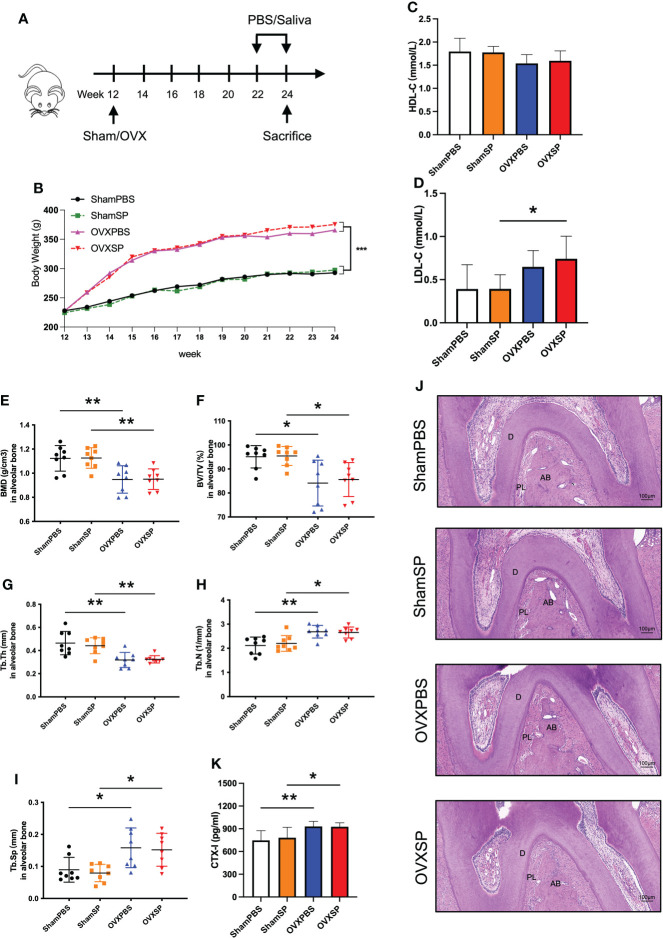
Effects of OVX and salivary microbiota treatment on alveolar bone. **(A)** Experimental schematic diagram. **(B)** Line chart of body weight change in rats. **(C)** HDL-C and **(D)** LDL-C levels in serum from the four groups. **(E–I)** Quantitative analysis of bone-related parameters. Compared to the Sham groups, the OVX groups exhibited significant trabecular bone loss at furcation area of the maxillary second molar, which showed decreased BMD **(E)**, BV/TV **(F)**, Tb.Th **(G)**, Tb.N **(H)**, and a higher Tb.Sp **(I)**. **(J)** Representative images of HE staining of alveolar bone. **(K)** Serum CTX-I levels measured using ELISA. Data are presented as the mean ± SD (n = 8/group). AB, alveolar bone; D, dentine; PL, periodontal ligament area. Scale bar = 100 μm. *p < 0.05 and **p < 0.01.

A schematic diagram is shown in **Figure 1A**. Body weight increased rapidly following OVX surgery (**Figure 1B**). Treatment with the periodontitis salivary microbiota alone showed no effect on the gain rates of body weight and HDL-C levels compared with PBS gavage (*p* > 0.05) (**Figure 1C**). LDL-C levels in the OVXSP rats were higher than those in the OVXPBS rats (*p* < 0.05). Neither HDL-C nor LDL-C showed statistically significant differences between the ShamPBS and ShamSP rats (**Figure 1D**).

To investigate the effects of OVX surgery and periodontitis salivary microbiota on the alveolar bone, we determined the microstructure of the trabeculae using micro-CT. The OVXPBS and OVXSP groups had a porous microarchitecture (**Supplementary Figure 2**), including a lower BMD (**Figure 1E**) and looser construction of more widely separated trabeculae than the ShamPBS and ShamSP groups (**Figures 1F–I**). HE staining revealed that the area of the periodontal ligament increased in the root furcation of OVX rats (**Figure 1J**), suggesting that the interradicular bone mass reduced as a result of OVX surgery. Furthermore, the serum CTX-I levels were significantly increased after OVX (*p* < 0.05) (**Figure 1K**); thus, an osteoporotic phenotype was successfully established in the alveolar bone. Nevertheless, no significant changes in alveolar bone were observed after intragastric administration of PBS or periodontitis salivary microbiota (**Figures 1E–J**).

### Salivary microbiota of periodontitis patients aggravates long bone loss in OVX rats

**Figure 2:**
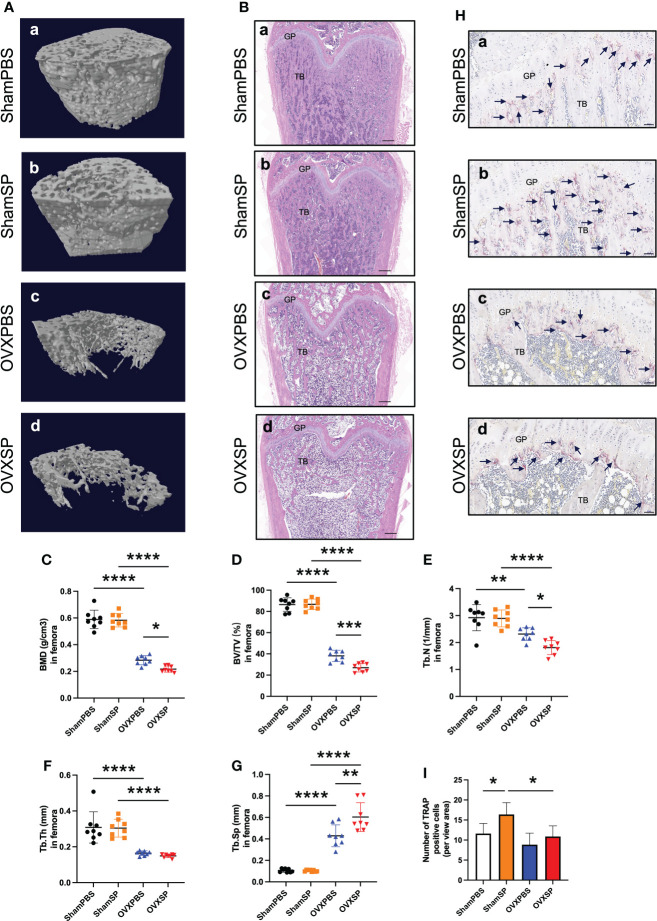
Effects of OVX and salivary microbiota treatment on the femur. **(A)** Representative reconstruction of trabeculae in the femurs used for analysis. **(B)** Representative images of the HE-stained femurs. Scale bar = 500 μm. **(C–G)** Quantitative analysis of bone-related parameters. Compared to the OVXPBS group, the OVXSP group had a significant decrease in bone mass and impaired bone microstructure; **(C)** BMD, **(D)** BV/TV, **(E)** Tb.N, **(F)** Tb.Th, and **(G)** Tb.Sp (n = 8/group). **(H, I)** Immunohistological analysis of TRAP staining (n = 6/group). Arrows indicate TRAP+ cells. Scale bar = 50 μm. Data are presented as the mean ± SD. GP, growth plate; TB, trabecular bone. *p < 0.05, **p < 0.01, ***p < 0.001, and ****p < 0.0001.

Micro-CT analysis and HE staining validated an osteoporotic phenotype in the femurs of OVX rats (**Figures 2A, B**). Furthermore, the periodontitis salivary microbiota gavage led to a significant decrease in bone mass and impaired bone microstructure of the femurs in OVX rats, as indicated by lower BMD, BV/TV, and Tb.N, and a higher Tb.Sp, compared to the OVXPBS group; in contrast, no difference was observed between the ShamPBS and ShamSP groups (**Figures 2C–G**). Histological analysis showed that the trabecular bone area of the femur in the OVXSP group was markedly smaller than that in the OVXPBS group (**Figure 2B**). Osteoclasts were almost absent in the trabecular region, except in the growth plate, of the femur in OVX rats (**Figure 2H**), suggesting that the destruction of trabeculae had peaked. The largest number of osteoclasts was observed in the ShamSP group (**Figures 2H, I**). These results suggest that the salivary microbiota of patients with periodontitis aggravates long bone loss, particularly in OVX rats.

### OVX and salivary microbiota affect intestinal inflammation

**Figure 3:**
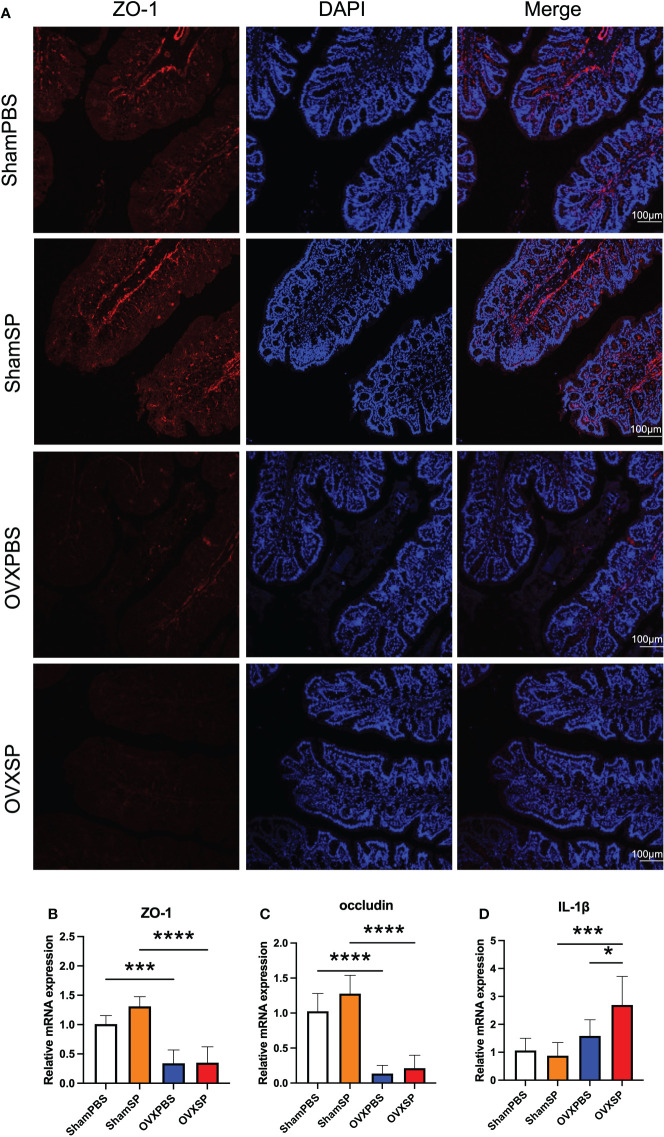
Effects of OVX and salivary microbiota treatment on intestinal permeability and inflammation. **(A)** Immunofluorescence analysis of ZO-1 expression (ZO-1, red; nucleus, blue) in colon. Scale bar = 100 μm. **(B–D)** mRNA levels of ZO-1 **(B)**, occludin **(C)**, and IL-1β **(D)** were measured using RT-qPCR. Data are presented as the mean ± SD (n = 5 to 6/group). *p < 0.05, ***p < 0.001, and ****p < 0.0001.

To determine whether the periodontitis salivary microbiota affects intestinal barrier integrity, the expression of tight junction (TJ) proteins was analyzed. Immunohistochemical staining showed lower ZO-1 expression in the colon of OVX rats (**Figure 3A**), while the transcription of ZO-1 and occludin was also downregulated in these rats (**Figures 3B, C**). No significant difference in ZO-1 or occludin was observed after the periodontitis salivary microbiota treatment in this experiment. The OVX rats exhibited a higher IL-1β mRNA expression; moreover, the expression of IL-1β mRNA was significantly elevated after gavage with the periodontitis salivary microbiota (*p* < 0.05) (**Figure 3D**). The above results indicate that OVX may impair intestinal barrier integrity; furthermore, OVX and periodontitis salivary microbiota may have a synergistic effect in promoting intestinal inflammation.

### Salivary microbiota alters the microbiota composition

**Figure 4:**
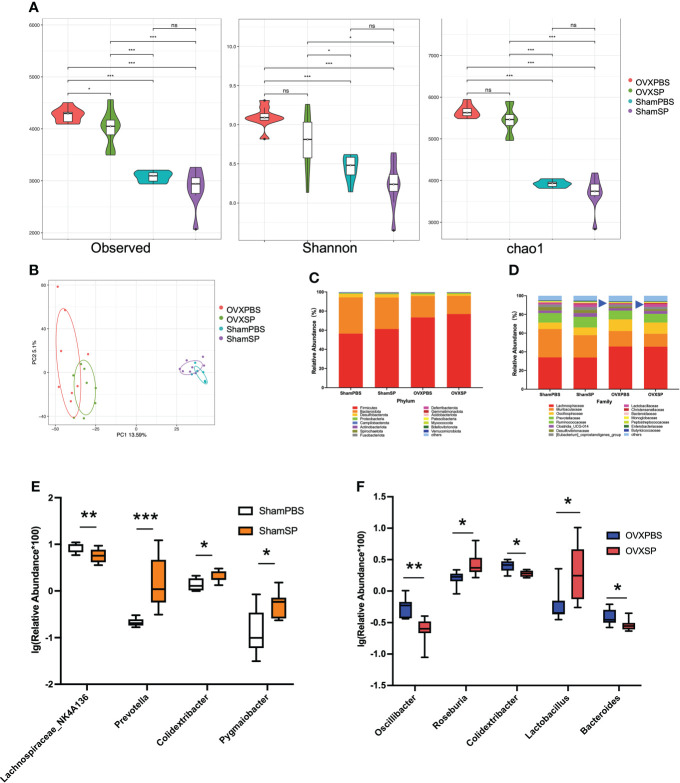
The salivary microbiota alters the gut microbiota. **(A)** Violin Plots of alpha diversity estimated by Observed, Shannon, and Chao1. **(B)** PCA of the cecum microbiota. **(C, D)** Relative abundance of gut microbiota at the phylum **(C)** and family **(D)** levels. **(E, F)** Relative abundance of the main differential gut microbiota combined with random forest at the genus level in Sham rats **(E)** and OVX rats **(F)**. The relative abundance was transformed by logarithm (log_10_). Boxplots and whiskers show the median, quantiles, and 1.5 interquartile ranges (n = 8/group). *p < 0.05, **p < 0.01, and ***p < 0.001.

Previous studies have demonstrated crosstalk between the gut microbiota and bone metabolism. In order to reveal the possible mechanism of the oral-gut axis in treatment with salivary microbiota of patients with periodontitis, alterations in the gut microbiota were analyzed by detecting the 16S rRNA gene. The indices of α diversity (Observed, Shannon, Chao1) in OVX rats were significantly higher than those in Sham rats (*p* < 0.05; **Figure 4A**). Furthermore, the salivary microbiota of periodontitis slightly reduced the observed species diversity in OVX rats but not in Sham rats, which is similar to the trabecular phenotype of the femur. Principal component analysis (PCA) revealed distinct clustering of the gut microbiota composition in the four groups (**Figure 4B**). The ratio of *Firmicutes* to *Bacteroidota*, a known marker of obesity, was elevated in OVX rats (**Figure 4C**), consistent with the changes in weight and LDL-L. At the family level, the OVXSP group possessed a significantly higher relative abundance of *Lactobacillaceae* than the OVXPBS group (*p* < 0.05; **Figure 4D**). To further identify the gut microbial composition affected by the salivary microbiota of patients with periodontitis, the relative abundance of the gut microbiota at the genus level was compared. The abundance of 77 genera (29 genera in Sham rats and 48 genera in OVX rats) significantly changed in response to treatment with periodontitis salivary microbiota (**Supplementary Figures 3A, B**). We then integrated the differential gut microbiota using random forest analysis (**Supplementary Figures S3C, D**). Specifically, after gavage with saliva, a significant increase in *Prevotella, Colidextribacter and Pygmaiobacter* abundances, as well as a decrease in that of *Lachnospiraceae_NK4A136*, were detected in the Sham rats (**Figure 4E**). However, the salivary microbiota exhibited a completely different effect on the gut microbiota composition in OVX rats. The OVXSP group presented higher amounts of *Roseburia* and *Lactobacillus* and lower relative abundances of *Oscillibacter, Colidextribacter*, and *Bacteroides* than the OVXPBS group (**Figure 4F**).

### Salivary microbiota affects serum metabolites

**Figure 5:**
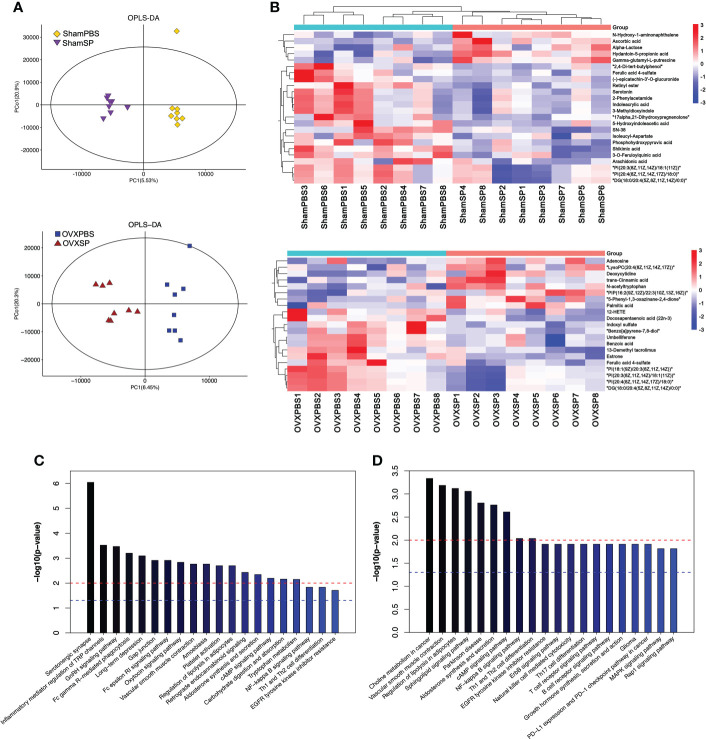
Effect of salivary microbiota on serum metabolites. **(A)** OPLS-DA plot of serum metabolites. **(B)** Heatmap showing the differential metabolites by salivary microbiota gavage. **(C, D)** Pathway analysis of the top 20 differentially enriched metabolites after gavage in Sham groups **(C)** and OVX groups **(D)**.

Serum metabolic profiles are shown in an OPLS-DA plot (**Figure 5A**) and were notably separated from each other, indicating that the salivary microbiota of patients with periodontitis has a profound effect on the serum metabolic profiles. Hierarchical clustering was conducted for differential metabolites—variables with *p* < 0.05 and VIP > 1.0. Notably, the differential metabolites influenced by the periodontitis salivary microbiota were enriched for lipids, indoles, and their derivatives (**Figure 5B**). The differential metabolic pathways identified by KEGG analysis are shown in **Figures 5C, D**. In Sham rats, serotonergic synapse, inflammatory mediator regulation of TRP channels, and the GnRH signaling pathway were the top three pathways. In OVX rats, the most different pathways between OVXPBS and OVXSP groups were choline metabolism in cancer, vascular smooth muscle contraction, and the regulation of lipolysis in adipocytes. Serotonin, indole, and derivatives are tryptophan metabolites. Given the above, these findings suggest that metabolism related to lipolysis and tryptophan might play an important role in aggravating bone loss.

### Correlations among parameters of bone, serum metabolites, and gut microbiota composition

**Figure 6:**
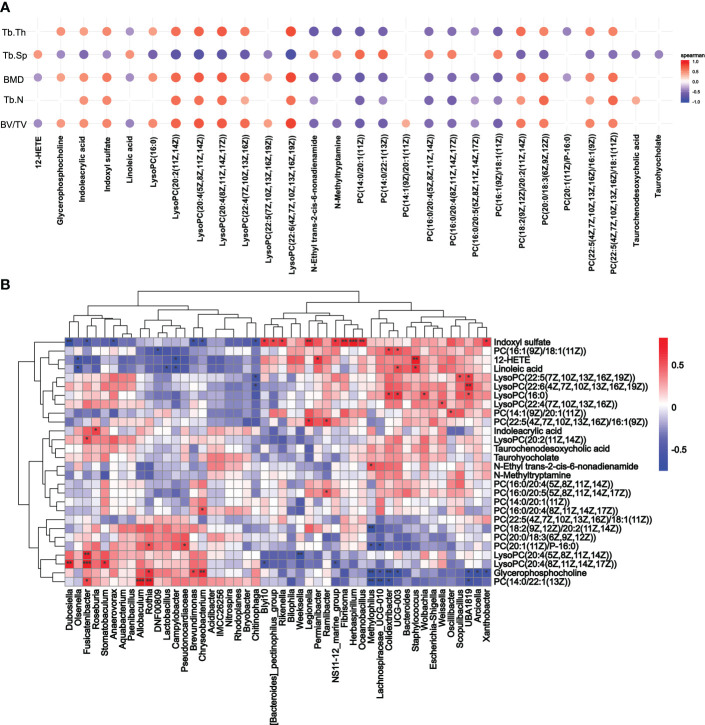
Correlation analysis of metabolites. **(A)** Spearman’s rank correlation analysis of the femoral parameters (BMD, BV/TV, Tb.Th, Tb.N, and Tb.Sp) and serum metabolites (lipids, lipid-like molecules, indoxyl sulfate, indoles and derivatives). Only *p* < 0.05 and correlations with a correlation coefficient greater than 0.1 are presented. **(B)** Correlation between metabolites screened from **(A)** and changes in genus abundance of OVX rats. *p < 0.05, **p < 0.01, and ***p < 0.001.

As the differential metabolic profiling pointed to lipids, indole, and tryptophan metabolites, Spearman’s rank correlation analysis was conducted for bone parameters (BMD, BV/TV, Tb.Th, Tb.N, and Tb.Sp) and metabolites (lipids, lipid-like molecules, indoxyl sulfate, indoles and derivatives). A total of 28 serum metabolites were identified (**Figure 6A**). We then explored the potential involvement of the differential gut microbiota (48 genera) in mediating the metabolites induced by the periodontitis salivary microbiota in OVX rats (**Figure 6B**). Indoxyl sulfate, known as tryptophan metabolite catabolism by the gut microbiota, was significantly correlated with 15 genera. Glycerophosphocholine was significantly correlated with 10 genera. PC (14:0/22:1(13Z)) was significantly correlated with 7 genera. LysoPCs, 12-HETE, linoleic acid, indoleacrylic acid, N-ethyl trans-2-cis-6-nonadienamide, and other phosphatidylcholines were significantly correlated with no more than 5 genera. A total of 7 metabolites and 10 genera were not significantly correlated with any other genera or metabolites.

## Discussion

Periodontitis and osteoporosis are prevalent conditions characterized by bone resorption. The consensus report of the 2017 World Workshop concluded that postmenopausal osteoporosis can increase alveolar bone turnover leading to net bone loss (Albandar et al., 2018; Jepsen et al., 2018). To further explore the relationship between periodontitis and osteoporosis, a bilateral ovariectomy was performed in our study. We successfully established a postmenopausal osteoporosis rat model, and the alveolar bone density was reduced significantly in OVX rats compared to Sham rats, consistent with our previous study (Luo et al., 2014).

The periodontal pocket provides a convenient channel for periodontal pathogens and inflammatory cytokines to enter the circulation, which is considered a major mediator of osteoporosis and periodontitis (Yu and Wang, 2022). To avoid the influence of the periodontal pocket, the rats were gavaged with the salivary microbiota of periodontitis, instead of the maxillary molar ligation. In the femoral neck, periodontal salivary microbiota led to a marked decrease in BMD and an impaired bone microstructure in OVX rats; nevertheless, no significant changes were observed in the alveolar bone. Earlier reports found that bone loss in the femur occurred 4 weeks after OVX (Li et al., 1997); however, it was not until 9 weeks after OVX that the reduced alveolar bone volume was significant (Tanaka et al., 2002; Johnston and Ward, 2015), suggesting that the alveolar bone was much more resistant to bone resorption than the femur.

The intestinal epithelium is an essential barrier for pathogen resistance. TJ proteins play crucial roles in paracellular permeability. The expression and distribution of TJ proteins can be affected by both physiological and pathological stimuli, leading to increased paracellular permeability and facilitation of pathogen colonization (Konig et al., 2016). In our study, OVX induced the disruption of TJ proteins, consistent with previous studies (Jia et al., 2019; Li et al., 2020). This finding may explain why the periodontitis salivary microbiota aggravated long bone resorption only in OVX rats. Proinflammatory cytokines, which can stimulate osteoclastogenesis and induce bone loss, also participate in regulating intestinal barrier integrity (Xu et al., 2017; Wang et al., 2021; Qiao et al., 2022). Consistent with changes in the femoral phenotype, the IL-1β expression was significantly elevated in OVX rats after salivary gavage. Our previous study also showed that IL-1β was highly expressed in colitis mice after periodontitis salivary microbiota gavage (Qian et al., 2022b). Recent studies have shown that ectopic colonization of oral pathobionts into gut may contribute to the activation and/or expansion of memory T cells by inducing IL-1β expression (Kitamoto et al., 2020; Read et al., 2021). Moreover, the IL-1β expression of periodontitis patients was higher than healthy patients, according to previous reports (Park et al., 2014; Qian et al., 2022b). These results suggest that IL-1β is potentially involved in the bone loss induced by periodontitis salivary microbiota.

The α diversity of the gut microbiota was significantly increased in OVX rats. After salivary gavage, more differential bacteria were observed in the OVX groups than in the Sham groups, suggesting that the colonization resistance mediated by the gut microbiota was weakened in OVX rats. In our study, the proportions of genera, including *Oscillibacter, Roseburia, Colidextribacter, Lactobacillus*, and *Bacteroides*, were significantly altered; these genera are related to lipid and amino acid metabolism (Natividad et al., 2018; Liu et al., 2022; Yan et al., 2022). Interestingly, the family *Lactobacillaceae* and genus *Lactobacillus* were both increased in the OVXSP group. *Lactobacillus* is one of the most well-understood probiotics. Effector metabolites include short-chain fatty acids, tryptophan, bacteriocins, and lactic acid (Xiao et al., 2021). A previous study indicated an association between *Lactobacillaceae* and obesity (Li et al., 2013). The relative abundance of *Lactobacillus* was also increased in obesity and osteoporosis (Larsen et al., 2010; Li et al., 2013; Das et al., 2019; Huang et al., 2019). However, no significant correlation was observed between *Lactobacillus* and BMD; thus, it appears that the change in *Lactobacillus* might be due to a compensatory effect.

Excessive fat accumulation can increase intestinal permeability, which, in turn, could lead to bone metabolism disorders (Zhang Z. et al., 2021). Our study showed that nearly half of the differential metabolites between OVXPBS and OVXSP were lipids and lipid-like molecules. According to hierarchical clustering and Spearman’s rank correlation analysis, 12-HETE, indoxyl sulfate, and LysoPC(20:4(8Z,11Z,14Z,17Z)) may be associated with the bone loss induced by the salivary microbiota. The level of 12-HETE, a bioactive lipid derived from arachidonic acid, significantly decreased in the OVXSP group; however, the levels of LysoPCs, which reportedly have proinflammatory activity (Xia et al., 2020), were significantly increased in the OVXSP group. In addition, indoxyl sulfate, a chronic kidney disease-specific renal osteodystrophy metabolite, was substantially reduced in the OVXSP group. Indoxyl sulfate is a product of tryptophan, which is metabolized to indole by the gut microbiota. As previously reported, indoxyl sulfate can control bone cellular processes through aryl hydrocarbon receptor signaling (Liu et al., 2018). Short-term and low-dose indoxyl sulfate leads to osteoclast differentiation, whereas long-term and high-dose indoxyl sulfate inhibit osteoclast differentiation (Liu et al., 2020). Thus, the optimal concentration and duration of metabolites remain to be studied. Considering the changes in gut microbiota, periodontitis salivary microbiota aggravates bone loss in OVX rats, possibly through lipid and amino acid metabolism generated by gut microbiota.

Our study has several limitations. First, the two-week salivary gavage period was too short to detect changes in the jaw; therefore, it is necessary to extend the treatment duration in subsequent studies. Second, the potential pathways identified in our results need to be confirmed by further *in vitro* and *in vivo* studies. Nevertheless, our study demonstrated that the contribution of the oral-gut axis to periodontitis salivary microbiota aggravates bone loss. The salivary microbiota of patients with periodontitis can alter the composition of the gut microbiota and increase intestinal inflammation, leading to changes in serum metabolites. These results suggest a novel mechanism by which periodontitis affects systemic disease.

## Data availability statement

The data presented in the study are deposited in the NCBI SRA database, accession number PRJNA855534.

## Ethics statement

The studies involving human participants were reviewed and approved by Ethical Committee of the Nanjing Stomatological Hospital, Medical School of Nanjing University. The patients/participants provided their written informed consent to participate in this study. The animal study was reviewed and approved by ethics authorities of Nanjing Agriculture University.

## Author contributions

FY designed and supervised the study. NW performed the majority of experiments and wrote the manuscript. YL, JQ, and LZ contributed to data interpretation and revised the paper. MW, LL, YH, and LZ provided technical assistance and assisted in the animal experiment. QZ and YL assist in data analysis. All authors contributed to the article and approved the submitted version.

## Funding

This project was supported by National Natural Science Foundation of China (81970939), the Nanjing Clinical Research Center for Oral Diseases (2019060009) and Nanjing Medical Science and Technology development fund (YKK21180).

## Acknowledgments

We thank Heng Dong, Qiang Li, Xiaoxin Zhang and Liang Ding at the Central Laboratory of Stomatology, Nanjing Stomatological Hospital, Medical School of Nanjing University for technical assistance. We also thank BioRender for providing drawing elements, which helped visualize our research.

## Conflict of interest

The authors declare that the research was conducted in the absence of any commercial or financial relationships that could be construed as a potential conflict of interest.

## Publisher’s note

All claims expressed in this article are solely those of the authors and do not necessarily represent those of their affiliated organizations, or those of the publisher, the editors and the reviewers. Any product that may be evaluated in this article, or claim that may be made by its manufacturer, is not guaranteed or endorsed by the publisher.
