# Supplementary material for: Salivary microbiota of periodontitis aggravates bone loss in ovariectomized rats
Source: Front Cell Infect Microbiol. 2022 Aug 12;12:983608. doi: 10.3389/fcimb.2022.983608 (PMC9411930; doi:10.3389/fcimb.2022.983608)
Supplement: Supplementary file 1 [file DataSheet_1.docx]

Supplementary Material

Table S1: List of primers used for qPCR

| Gene | Forward primer | Reverse primer |
| --- | --- | --- |
| ZO-1 | CACCACAGACATCCAACCAG | CACCAACCACTCTCCCTTGT |
| Occludin | TCTCAGCCGGCATACTCTTT | ATAGGCTCTGTCCCAAGCAA |
| IL-1β | CACCTCTCAAGCAGAGCACAG | GGGTTCCATGGTGAAGTCAAC |
| β-actin | CACCCGCGAGTACAACCTTC | CCCATACCCACCATCACACC |


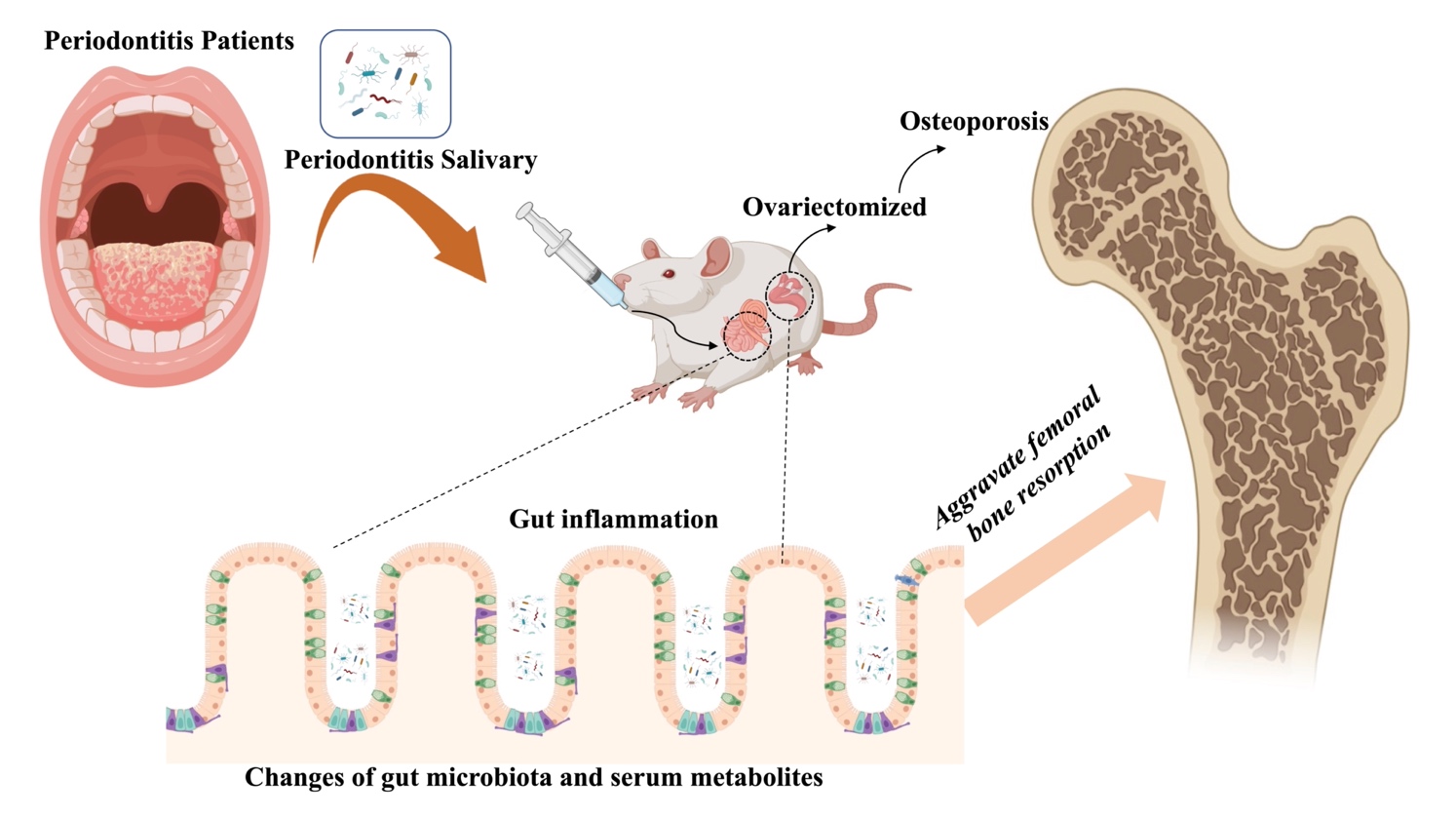


**Supplementary Figure 1.** **Schematic diagram**. The salivary microbiota of patients with periodontitis can aggravate long bone resorption in ovariectomized rats through oral-gut axis.


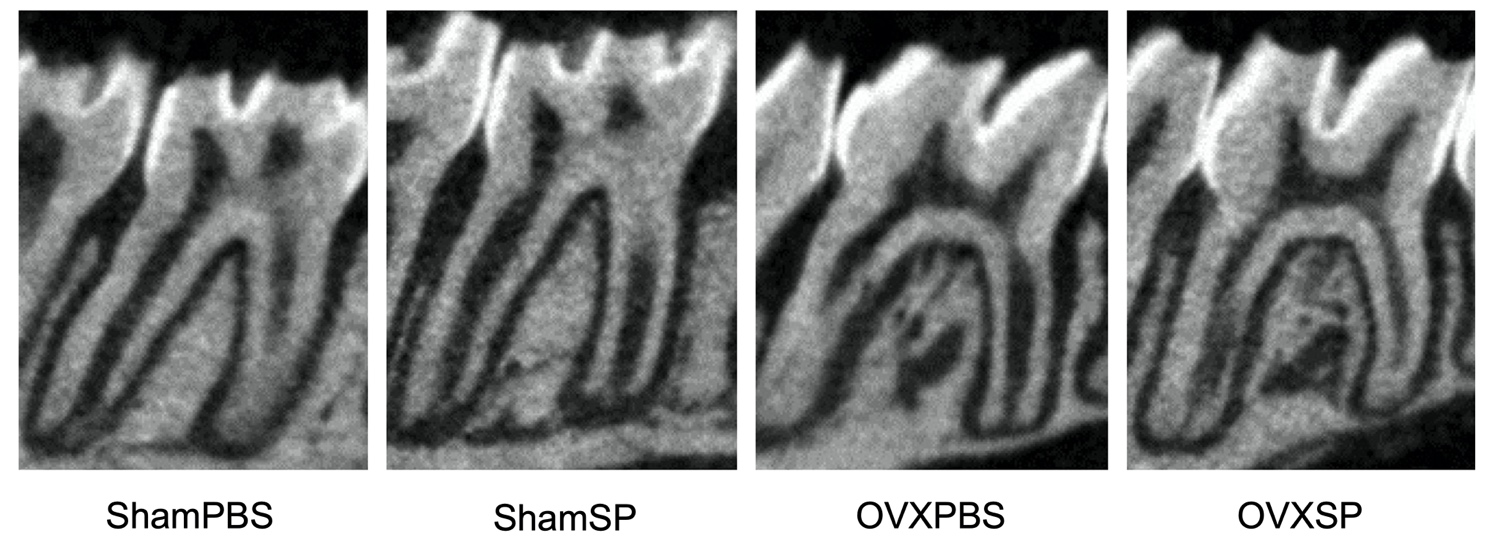


**Supplementary Figure 2.** **Representative micro CT images of** **the root furcation area of maxillary second molar**. Compared to the ShamPBS and ShamSP groups, the OVXPBS and OVXSP groups had a porous microarchitecture.


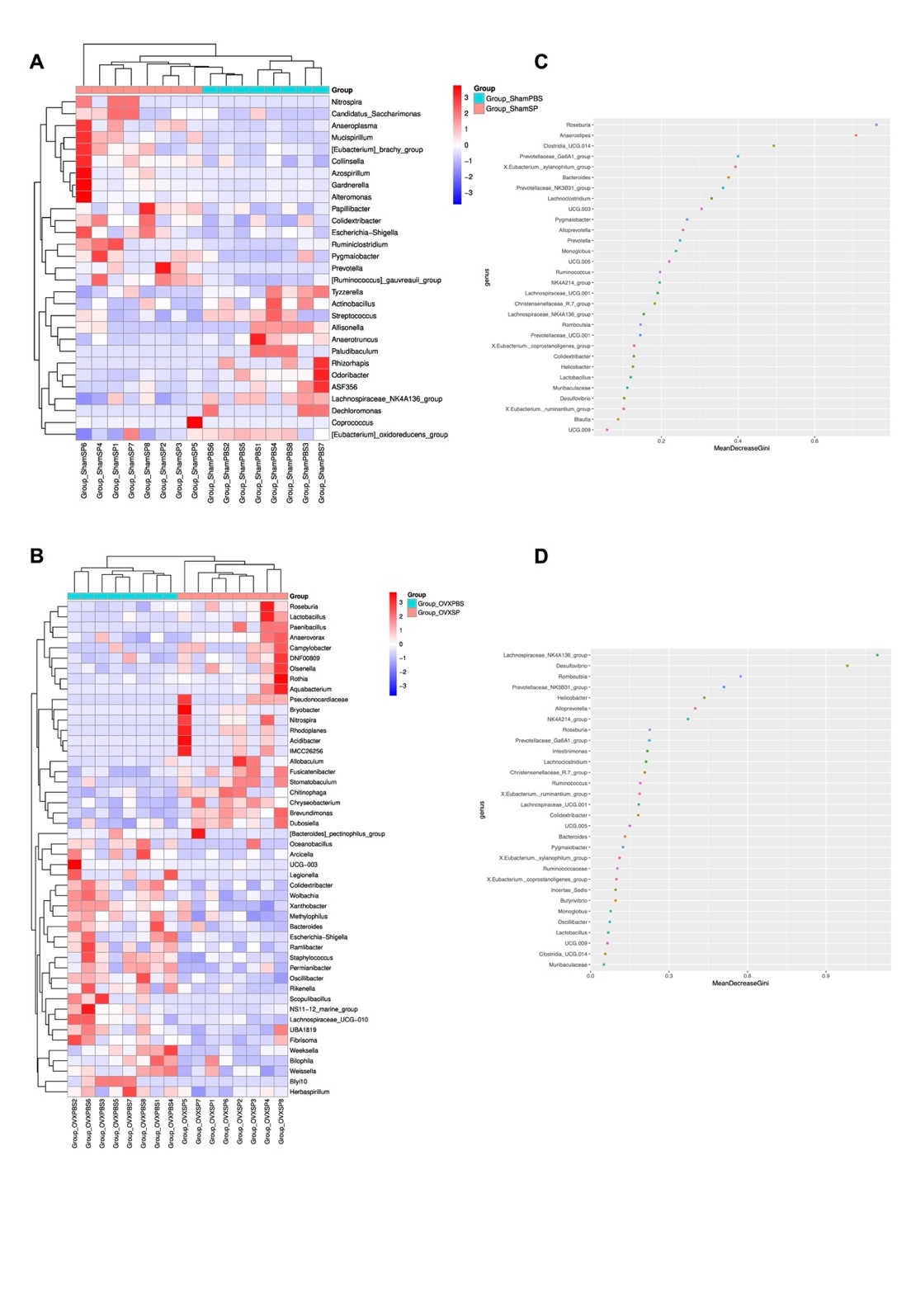


**Supplementary Figure 3.** **Effects of salivary microbiota treatment on gut microbiota.** **(A–B)** Heatmap showing the differential microbiota at genus level in Sham rats **(A)** and OVX rats **(B)**. **(C–D)** Random Forest analysis of gut microbiota in Sham rats **(C)** and OVX rats **(D)**.
